# Targeted alpha therapy using short-lived alpha-particles and the promise of nanobodies as targeting vehicle

**DOI:** 10.1080/14712598.2016.1185412

**Published:** 2016-05-19

**Authors:** Yana Dekempeneer, Marleen Keyaerts, Ahmet Krasniqi, Janik Puttemans, Serge Muyldermans, Tony Lahoutte, Matthias D’huyvetter, Nick Devoogdt

**Affiliations:** ^a^ Vrije Universiteit Brussel, In Vivo Cellular and Molecular Imaging, Brussels, Belgium; ^b^ Nuclear Medicine Department, UZ Brussel, Brussels, Belgium; ^c^ Vrije Universiteit Brussel, Laboratory of Cellular and Molecular Immunology, Brussels, Belgium

**Keywords:** Cancer, targeting vehicles, targeted alpha therapy, radionuclide labeling, nanobody, bismuth-213, astatine-211

## Abstract

**Introduction**: The combination of a targeted biomolecule that specifically defines the target and a radionuclide that delivers a cytotoxic payload offers a specific way to destroy cancer cells. Targeted radionuclide therapy (TRNT) aims to deliver cytotoxic radiation to cancer cells and causes minimal toxicity to surrounding healthy tissues. Recent advances using α-particle radiation emphasizes their potential to generate radiation in a highly localized and toxic manner because of their high level of ionization and short range in tissue.

**Areas covered**: We review the importance of targeted alpha therapy (TAT) and focus on nanobodies as potential beneficial vehicles. In recent years, nanobodies have been evaluated intensively as unique antigen-specific vehicles for molecular imaging and TRNT.

**Expert opinion**: We expect that the efficient targeting capacity and fast clearance of nanobodies offer a high potential for TAT. More particularly, we argue that the nanobodies’ pharmacokinetic properties match perfectly with the interesting decay properties of the short-lived α-particle emitting radionuclides Astatine-211 and Bismuth-213 and offer an interesting treatment option particularly for micrometastatic cancer and residual disease.

## 1. Introduction

### 1.1. Targeted radionuclide therapy

The evolution of modern medicine during the second half of the twentieth century has improved the clinical outcome of patients with numerous forms of cancer. Today, the treatment of cancer generally consists of surgery, systemic chemotherapy, radiation therapy (including external beam radiation), immunotherapy, antihormone therapy, targeted radionuclide therapy (TRNT). The choice depends upon the location and grade of the tumor and the stage of the disease, as well as the general state of the patient. Presently, tumor reduction by chemotherapy is increasingly being used in combination with surgery in multiple cancer types. Chemotherapy interacts with vital processes of the cell cycle or cell metabolism, thereby stopping or reversing cancer growth. Chemotherapy does not distinguish cancer cells from certain healthy cells, making it a less specific treatment option. External beam radiation is not suited for disseminated disease and immunotherapy often has to deal with specific resistance issues.[1]

The main objective of TRNT is the ability to selectively deliver cytotoxic radiation to cancer cells that causes minimal toxicity to surrounding healthy tissues, using optimized vehicles that deliver a nuclear payload into the tumor cells. TRNT is a growing and favorable treatment option for cancer. Currently, two principal categories can be distinguished. First, there are agents that accumulate naturally in tumor tissue. Examples are Iodine-131 (^131^I) for the treatment of differentiated thyroid cancer [2] and Strontium-89 (^89^Sr) and Radium-223 (^223^Ra) for the treatment of bone metastases.[3,4] ^131^I and ^89^Sr are both β^−^-particle-emitting radionuclides, while ^223^Ra is an α-particle-emitting radionuclide. The second category includes agents that target tumor-associated antigens that are aberrantly present in malignant tissue. Examples are Yttrium-90 (^90^Y)- and Lutetium-177 (^177^Lu)-octreotide as radiolabeled peptides to treat somatostatin-overexpressing neuroblastoma.[5–7] In addition, monoclonal antibodies (mAbs) are also used as vehicles to target tumor-associated antigens and hereby providing a specific internal radiotherapy.[8] The only regulatory-approved radiolabeled mAb is ^90^Y-ibritumomab to treat non-Hodgkin lymphoma.[9,10]

Thus, a radiopharmaceutical usually consists of two parts: a targeting biomolecule that specifically determines the localization of the radiopharmaceutical and a radionuclide that delivers the mechanism of action through its decay. Today, radiopharmaceuticals are used as either diagnostics for noninvasive imaging through the detection of γ-rays using positron emission tomography (PET) or single-photon emission computerized tomography (SPECT), and/or as therapeutics to deliver radiation to the targeted tumor cells. When radiopharmaceuticals are employed both for diagnosis and therapy, they are referred to as ‘theranostic agents.’ This combined diagnostic–therapeutic procedure uses a diagnostic test to determine whether a patient may benefit from a specific therapeutic drug, allowing personal, structural, and functional characterization of a tumor during therapy. Moreover, the therapy response could be measured throughout the therapy.

In general, there are three types of radiation that can be used for TRNT: β^−^-particles, Auger electrons, and α-particles. Each radionuclide is characterized by its own decay properties, tissue range, half-life, and chemistry, proposing the opportunity to adapt the features of the radionuclide to a particular type of cancer and in the long run to the needs of an individual patient.[11] Until now, TRNT has been mainly explored using β^−^-particle-emitting radionuclides. β^−^-Particles have a low linear energy transfer (LET) (0.2 keV/μm), producing repairable DNA damage including single- or double-stranded DNA breaks, base chemical modifications, and protein crosslinks. In case of low-LET radiation, like for example β^−^-particles, the damage caused by direct ionization of the target might only be sublethal, if dosed insufficiently high. Indirect effects caused by reactive oxygen species (ROS) also contribute to the eventual damage. β^−^-Particles have a relatively long range in tissue (1–10 mm), causing cytotoxic damage in surrounding nontargeted cells, referred to as ‘crossfire effect.’ This might be useful for the treatment of heterogeneous, bulky tumors, but it has the disadvantage of damaging surrounding normal tissue. Most progress with β^−^-particle radiation has been made in hematological malignancies, while the progress in epithelial-derived tumors has been slow. One of the shortcomings of low-LET β^−^-particle-emitters is that much more of the radioactivity need to reach the tumor tissue to effectively kill it, compared to high-LET α-particles. A single α-particle is sufficient to destroy the cell nucleus, as cell death due to the α-radiation is largely independent of oxygenation or active cell proliferation. β^−^-Particles on the other hand need much more hits at the level of the cell nucleus as they produce sparse ionization and individual DNA lesions, mostly repairable. This disadvantage is one of the reasons for the average success of agents labeled with β^−^-particle-emitting radionuclides in clinical trials. Theoretically, Auger-electron emitters present multiple advantageous characteristics, making it an attractive candidate for TRNT. Auger emitters have a short effect range (subcellular, order of nanometers), a LET of 4–26 keV/μm, and are able to produce a high level of cytotoxicity due to Auger electron cascades. These cascades, by which electrons, carrying a characteristic kinetic energy, are ejected from atoms in response to a downward transition by another electron in the atom. In contrast to α-radiation, Auger radiation is of low toxicity when decaying outside the cell nucleus, such as in the cytoplasm or outside of cells, and will therefore cause little damage to nontargeted cells. Some studies have shown that Auger electrons can be effective when targeted only to the cell membrane.[12] However, it is generally considered that the radioisotope needs to be delivered close to the cell nucleus in order to be effective, which makes internalization into the cell crucial.[13]

### 1.2. General considerations of targeted alpha therapy

The selection of the appropriate radionuclide depends on its decay properties, namely the physical half-life and emission characteristics. For the management of bulky and heterogeneous tumors, treatment with β^−^-particle-emitting radionuclides might be the preferred approach. However, for the eradication of small-volume tumors and small clusters of cancer cells, agents that emit high-energy α-particles would be more beneficial due to their highly specific toxic load to the targeted tumor cells and their short range in tissue. Thus, the main strength of targeted alpha therapy (TAT) is the potential to deliver radiation in a highly localized and toxic manner, because of their high level of ionization produced and short range in tissue.[14] An α-particle consists of a ^4^He nucleus; therefore, it is much heavier than other subatomic particles emitted from decaying radionuclides and nuclear reactions. The main characteristics of currently available α-particle-emitting radionuclides are summarized in Table 1.[12] With a charge of +2, α-particles are effective ionization agents with a high LET (50–230 keV/μm) at a short range of 50–100 μm in tissue. They induce clusters of DNA damage such as double-stranded DNA breaks and base chemical modifications that evoke a large number of cellular responses and pathways that include apoptosis, autophagy, necrosis, and cell-cycle arrest. This type of damage is difficult to repair by the cell. Moreover, the damage is independent from the generation of indirect ROS, leaving their effectiveness potentially unabated by tumor hypoxia.[12] These characteristics make α-emitters effective in eradicating small clusters or isolated cancerous cells with little exposure to surrounding healthy tissue. Thus, TAT is of high interest for the treatment of micrometastatic and minimal residual disease after surgery. Moreover, the concept of TAT has moved from bench to bedside, with increasing clinical experience in, for example, ovarian cancer, metastatic prostate cancer, gliomas, and acute myeloid leukemia (Table 2). A median survival of 8.9 months could be achieved after intravenous administration of the α-immunoconjugate, Bismuth-213 (^213^Bi)-cyclic diethylenetriaminepentaacetic acid anhydride (cDTPA)-9.2.27, in patients with metastatic melanoma in a phase I trial.[15] Using TAT to treat metastatic melanoma, α-particles reach the endothelial cell nuclei, causing cell death and leading to capillary closure and interruption of nutritional support to the tumor. If enough capillaries are closed down, the tumor might regress and could even disappear. Thus, this subtype of TAT targets specifically the vasculature and has been referred to as ‘tumor anti-vascular α-therapy (TAVAT).’[16] TAT has been compared to β^−^-particle-emitting radionuclides in several clinical trials, highlighting their promising therapeutic potential. For example, investigators compared ^131^I-labeled bisphosphonates with their Astatine-211 (^211^At)-labeled counterparts for pain relief in patients with bone metastasis.[17] In addition, Henriksen et al. explored the bone-seeking properties of ^223^Ra and compared it with those of the β^−^-particle-emitting radionuclide ^89^Sr.[3] The conclusion of both studies was that α-particle radiation showed a lower toxic effect to the healthy bone marrow compared to β^−^-particle emitters, which is attributed to the reduced crossfire effect. This and other studies indicated that the strength and short distance of high-LET α-particles make them more suitable than low-LET β^−^-particles in particular circumstances. Despite its positive features, the translation of TAT into the clinic has been slow, mainly due to the limited radionuclide availability and the short physical half-life and daughter α-particles of some of the available α-emitters. Furthermore, several other issues concerning α-particle emitters should be addressed as well, which are discussed in the following paragraphs.

**Table 1.** Main characteristics of the currently available α-particle-emitting radionuclides.

Isotope	Daughter isotopes*	Physical half-life	Maximum energy (keV)	Occurrence (%)	Associated emissions
^211^At	–	7.2 h	5.867	α (41.8%)	α, γ, LEE
	^211^Po	516 ms	7.450	α (100%)	
^225^Ac	–	10 days	5.830	α (100%)	α, γ, Auger, β^−^
	^221^Fr	4.9 min	6.341	α (100%)	
	^217^At	32.3 ms	7.069	α (99.98%)/β^−^ (0.01%)	
	^213^Bi	45.6 min	6.051	α (2.2%)/β^−^(97.8%)	
	^213^Po	4.2 µs	8.377	α (100%)	
^213^Bi	–	45.6 min	6.051	α (2.2%)/β^−^ (97.8%)	α, γ, Auger, β^−^
	^213^Po	4.2 µs	8.377	α (100%)	
^212^Bi	–	61 min	5.870	α (36%)/β^−^ (64%)	α, γ, Auger, β^−^
	^212^Po	298 ns	8.785	α (100%)	
^227^Th	–	18.72 days	6.038	α (100%)	α, γ, Auger, β^−^
	^223^Ra	11.4 days	5.871	α (100%)	
	^219^Rn	4 s	6.819	α (100%)	
	^215^Po	1.8 ms	7.386	α (100%)	
	^211^Bi	2.14 min	6.623	α (99.7%)/β^−^ (0.3%)	
^212^Pb	–	10.64 h		β^−^ (100%)	β^−^
	^212^Bi	61 min	5.870	α (36%)/β^−^ (64%)	α, γ, Auger, β^−^
	^212^Po	0.3 μs	8.785	α (100%)	
^223^Ra	–	11.4 days	5.871	α (100%)	α, γ, Auger, β^−^
	^219^Rn	4 s	6.819	α (100%)	
	^215^Po	1.8 ms	7.386	α (100%)	
	^211^Bi	2.14 min	6.623	α (99.7%)/β^−^ (0.3%)	

*Generated α-particle emitter after decay of the conjugated parent. LEE: Low-energy electron emission; NS: yield not significant.

**Table 2.** Vehicles used in targeted α-particle therapy in preclinical and clinical settings.

Radionuclide	TAT agent	Indication	Antigen	Reference (preclinical data)	Reference (clinical phase)
^225^Ac	Anti-CD33 IgG (HuM195)	Leukemia	CD33	[18]	I [19,20]
^225^Ac	Anti-HER2 IgG (trastuzumab)	Ovarian cancer	HER2	[21]	–
^227^Th	Anti-HER2 IgG (trastuzumab)	Breast and ovarian cancer	HER2	[22,23]	
^227^Th	Anti-CD20 IgG (rituximab)	Non-Hodgkin lymphoma	CD20	[24,25]	
^213^Bi	Anti-CD33 IgG (HuM195)	Leukemia	CD33	[26,27]	I and I/II [28,29]
^213^Bi	Anti-CD20 IgG (rituximab)	Non-Hodgkin lymphoma	CD20	[30,31]	I [32]
^213^Bi	Plasminogen activator inhibitor type 2	Breast cancer, pancreatic cancer	Urokinase plasminogen activator receptor	[33–35]	
^213^Bi	Anti‐MUC1 IgG (C595 IgG)	Ovarian cancer, pancreatic cancer	MUC1	[36,37]	
^213^Bi	Substance P	Glioblastoma	Neurokinin type-1 receptor		0/I [38,39]
^213^Bi	Anti-NG2 IgG (9.2.27 IgG)	Melanoma	NG2 proteoglycan	[40,41]	I [15,42,43]
^213^Bi	Anti-CD138 IgG	Multiple myeloma	CD138	[44]	
^213^Bi	Anti-PSMA IgG (J591 IgG)	Prostate cancer	PSMA	[45]	
^213^Bi	C6.5K-A scFv, C6.5K-A diabody	Breast and ovarian carcinomas	HER2	[46]	
^212^Pb/^212^Bi	Anti-HER2 IgG (TCMC-trastuzumab)	Ovarian cancer	HER2	[47,48]	[48–50]
^211^At	Chimeric 81C6 IgG	Glioblastoma	Tenascin-C	[51,52]	II [53]
^211^At	MX35 F(ab′)_2_	Ovarian cancer	NaPi2b	[54]	I [55]
^211^At	Anti-FRA IgG (Mov18)	Ovarian cancer	Folate receptor alpha	[56]	
^211^At	Anti-EGFRvIII IgG	Glioblastoma	EGFRvIII	[57]	
^211^At	Anti-HER2 C6.5 diabody	Breast cancer	HER2	[58]	
^211^At	Z_HER2:342_ and (Z_HER2:4_)_2_ affibody molecules	Breast and ovarian carcinomas	HER2	[59]	
^223^Ra	^223^Ra-chloride	Skeletal breast and prostate cancer metastases	Hydroxyapatite	[60]	I–III [61,62]

NG2: Neural/glial antigen 2; PSMA: prostate-specific membrane antigen; EGFRvIII: epidermal growth factor receptor variant III.

#### 1.2.1. Radiolysis

Radiolysis is the dissociation of molecules by nuclear radiation. The magnitude of energy deposits by volume of α-particle emitters is two times greater than that of β^−^-emitters such as ^90^Y or ^131^I. Because of this, the potential impact of radiolysis effects when using α-particles is noticeably higher. Hence, the radiolabeling of certain vectors with an α-particle emitter using high levels of radioactivity while maintaining appropriate biological properties may be challenging.[51] Studies by Zalutsky et al. indeed emphasize the potential importance of radiolysis-mediated effects on the chemistry of α-particle-emitting radiopharmaceuticals and the need to evaluate their labeling chemistry and stability at high doses required for clinical use.[63,64]

#### 1.2.2. The radiation-induced biological bystander effect

The radiation-induced biological bystander effect (RIBBE) is a process whereby nontargeted healthy cells are damaged, not as a result of directly being hit by radiation, but via the radiation-induced death or stress of neighboring cells. As α-particle-emitting radionuclides have a range in tissue that is equivalent to only a few cell diameters, the physical crossfire effect will be limited. To date, the majority of studies of RIBBE have been performed *in vitro* using single-cell or multicellular systems *ex vivo* or in artificial three-dimensional human tissue systems. Boyd et al. demonstrated that cell death in adjacent cells after treatment with α-particle-emitting radionuclides might be enhanced via RIBBE.[65] Furthermore, evidence on the *in vivo* effectiveness of RIBBE has been limited, but new findings indicate that they may affect tumor development in susceptible mouse models. For example, Mancuso et al. demonstrated that DNA double strand breaks and apoptotic cell death could be induced by bystander responses in mouse cerebellum after X-ray exposure of the remainder of the body.[66] Mice were whole-body exposed or irradiated with individual cylindrical lead shields providing protection of heads. Whole-body-irradiated animals developed cerebellar tumors. A high percentage of mice (62%) died of aggressive disease by 23 weeks, with median survival of 14 weeks. Significantly, they also observed a remarkably increased medulloblastoma rate (39%) in lead shielded-irradiated mice, indicating that bystander effects are factual *in vivo* events with carcinogenic potential. However, the underlying mechanisms are incompletely characterized and it remains unclear how processes involving oxidative metabolism and stress-inducible proteins lead to (oxidative) DNA damage in bystander cells.[67]

#### 1.2.3. Distribution of recoil daughters in the body

Another important aspect that should be taken into account is the unstable bond of daughter isotopes upon α-decay due to the different chemical properties of the daughters. This could result in an immediate loss of the daughter atom from the chelating chemistry.[68] In addition, the recoil energy of the recoiling daughters is more than 1000 times higher than the binding energy of any chemical compound, which will lead to the rupture of the chemical bonds of the daughter atom with the targeting vehicle, as well as to the ionization of the surrounding medium. The released daughter isotopes that are often themselves α-emitters might cause substantial harm since they will no longer be bound to the targeting vehicle. Therefore, it is of utmost importance to study the fate of both mother and daughter isotopes. For instance, the biodistribution of the bone-targeting radiopharmaceutical ^223^Ra, which naturally targets the hydroxyapatite matrix in the bone, has been studied extensively *in vivo*.[3,69] Although the daughter isotopes are not intrinsically bone-seeking, the rapid cascade of α-particle-emitting daughters will deliver high doses to bone metastases. However, their short half-life appears to prevent them from causing major damage to healthy tissue. An *in vivo* study demonstrated that less than 2% of the daughters migrate away from the bone surface within 6 h after administration of ^223^Ra, and after 3 days, this number has dropped down to less than 1%.[3] Another example is the decay of actinium-225 (^225^Ac) with the formation of potentially disadvantageous radiotoxic daughter products such as ^213^Bi. It is critically important to reduce the redistribution of the daughter isotopes to nontarget tissues and to diminish systemic radiotoxic events. Therefore, the ^225^Ac ‘nanogenerator’ approach was designed in which the delivery system is engineered to be internalized into the targeted tumor cell.[70] McDevitt and colleagues demonstrated the ability to safely and efficiently use ^225^Ac as a potent tumor-selective generator in both established solid carcinomas and disseminated cancers.[71] Although these results were very promising, additional development of this modality is warranted to optimize the stability of the nanogenerator to maximize the retention of the tumor while avoiding uptake in healthy organs.

#### 1.2.4. Dosimetry

Radiation dosimetry is the measurement of the absorbed dose delivered by the ionizing radiation and provides a basis for understanding the effects and efficacy of different radiation-based treatments. One of the major impediments of TRNT is the heterogeneous distribution of the radiopharmaceutical in normal and tumor tissues. In the case of α-particle radiation, their short path length and high LET need to be taken into account, posing an enormous challenge on the methods needed for relevant dosimetry.[72] For high-LET irradiation, the effect of a single incident in the nucleus of the cell is so abundant that the variations in absorbed dose (specific energy) to the nucleus can be very large and therefore might be a misleading index of the biologic effect. The clinical quantification of the absorbed doses with the γ-camera is only able to give an estimate about the uptake of the radiopharmaceutical in whole organs and in macroscopic tumors, while quantification of absorbed doses in smaller compartments in organs or microscopic tumors is barely feasible. Thus, small-scale dosimetry or microdosimetry, which takes into account the stochastic nature of energy deposited in small targets, would generate improved dosimetric calculations for α-particle radiation. Due to the limited clinical experience with α-particles to date, unknown maximum tolerable doses in humans are the major issue in TAT. In mice, absorbed doses of α-particle radiation can be calculated in tissues at a macroscopic level (organs and substructures) using Monte Carlo techniques based on fundamental physical principles.[73,74] In addition to that, Bäck and colleagues developed the α-camera, which is a quantitative imaging technique developed to detect α-particles in tissues *ex vivo* at suborgan level, to get a better view on the biodistribution of internal α-radiation on a cellular level.[75] The high-resolution (35 μm or less) α-camera was able to measure the activity distribution on a cellular level by virtue of the short path length of α-particles, making it a promising tool in the evaluation of future TAT.

## 2. The current developments

### 2.1. A milestone for TAT: radium-223

Radium (Ra) and polonium (Po) were first described by Marie and Pierre Curie in 1898 while investigating the radioactive properties of a complex ore, which had radioactive emissions in excess. ^223^Ra and ^89^Sr are bone-targeting radiopharmaceuticals with hydroxyapatite (Ca_5_[PO_4_]_3_OH) as target, which is an essential component of the inorganic bone matrix. Ra, barium (Ba), Sr, and calcium (Ca) are all chemicals in the alkaline earth metal family on the periodic table and each will localize in the areas of osteoblastic metastases. ^223^Ra is currently the most commonly used radioisotope for medical therapeutics, showing an increased survival in patients with metastatic castration-resistant prostate cancer [61] and has a half-life of 11.4 days (Table 1). ^223^Ra is the first α-emitter approved by the US Food and Drug Administration.[76] In addition, ^223^Ra is the first α-particle-based therapy that results in pain relief and extends survival in patients with progressive castration-resistant prostate cancer and bone metastasis in the absence of visceral metastasis. Thus, ^223^Ra is naturally incorporated in areas of increased bone turnover in bone metastases.[77] More than 90% of patients with metastatic resistant prostate cancer have radiologic evidence of bone metastases. ^223^Ra dichloride has been evaluated in two phase I trials and three double-blind phase II trials. The phase III ALSYMPCA (Alpharadin in the Treatment of Patients With Symptomatic Bone Metastases in Castration-Resistant Prostate Cancer) trial showed an improved overall survival of 3 months and pain relief in patients with osseous metastasis.[61] The success of ^223^Ra as a therapeutic further stimulates TAT-based preclinical and clinical research. In a way, ^223^Ra could be considered as a game changer in nuclear medicine, as it might facilitate the future use of additional high-LET particle emitters.

### 2.2. Other promising α-particle-emitting radionuclides

Besides ^223^Ra, many other α-particle emitters have suitable characteristics for therapeutic applications (Table 2). ^211^At, ^213^Bi, lead-212 (^212^Pb)/bismuth-212 (^212^Bi), and ^225^Ac are the most frequently used α-particle-emitting radionuclides in clinical molecular targeting applications to date.[78]

#### 2.2.1. Actinium-225

^225^Ac is a parent α-particle emitter in a decay cascade that produces three net α-particle isotopes, ^221^Fr (half-life 4.8 min), ^217^At (half-life 32.3 ms), and ^213^Bi (half-life 45.6 min), making it a very effective and potent option for TAT (Table 1). ^225^Ac has a half-life of 10 days and can be produced by natural decay of ^233^U in Oak Ridge National Laboratory, USA [79] or by accelerator-based methods in Karlsruhe.[80] However, the latter production of ^225^Ac also results in the production of ^227^Ac which decays with a half-life of 21.772 years. The biggest disadvantage concerning ^225^Ac is its cost, which might reach to $1200/mCi. In addition, the recoiled daughters of ^225^Ac can do significant damage to healthy tissue when not retained at the tumor site. Encapsulation in a nano-carrier, fast uptake of the α-particle-emitting radionuclides in tumor cells, and local administration are some approaches to minimize toxic effects caused by α-particle-emitting daughters.[68] On the other hand, the relatively long half-life of ^225^Ac allows a centralized production and shipment of the irradiated targets to further users so that any investigator is able to exploit the power of this α-particle. Furthermore, ^225^Ac decays to ^213^Bi, of which the latter also results in a 440 keV γ-ray emission that can be useful for imaging of the therapeutic biodistribution. It should be remarked that it is uncertain whether the measured radioactive decay represents intact radiopharmaceutical or released daughter radioisotopes. Moreover, ^225^Ac can be conjugated to peptides or antibodies, using an optimized radiochemistry with standard widely available macrocyclic bifunctional chelators.[81,82] *In vivo* experiments showed that the ^225^Ac complex with 1,4,7,10-tetraazacyclododecane-*N*,*N*′,*N*′′,*N*′′′-tetra-acetic acid (DOTA) was more stable than the ^225^Ac complex with 4,7,10,13,16-hexaazacyclohexadecane- *N,N*′,*N*′′,*N*′′′,*N*′′′′,*N*′′′′′-hexaacetic acid.[70] The biodistribution aspects of ^225^Ac-labeled mAbs and other carriers, together with their pharmacokinetic properties, radiobiology, and dosimetry, have been reviewed by Miederer et al.[70] A successful phase I trial has demonstrated that a humanized anti-CD33 mAb HuM195 conjugated to ^225^Ac (Actimab-A) is safe to use at doses ≤0.1 MBq/kg [19] (Table 2).

#### 2.2.2. Bismuth-213

^213^Bi is most often produced through an ^225^Ac-generator. The principal drawbacks of using ^213^Bi are its very short physical half-life of 46 min and limitations regarding availability and cost as for ^225^Ac. Pippin and colleagues were the first to label ^213^Bi with mAbs.[83] Moreover, McDevitt and colleagues labeled ^213^Bi via the bifunctional metal cDTPA complex with a humanized mAb (HuM195) directed against CD33, a glycoprotein expressed on the majority of myeloid leukemia cells.[26] In subsequent studies, the stability of this radiopharmaceutical has been improved to achieve a clinically applicable ^213^Bi-CHX-A-DTPA-HuM195.[84] A phase I clinical study on 18 patients with acute myelogenous leukemia (AML) or chronic myelomonocytic leukemia showed no significant extramedullary toxicity, although myelosuppression was seen in all patients.[28] The phase I/II trials showed that sequential administration of cytarabine and ^213^Bi-CHX-A-DTPA-HuM195 was reported to be tolerable and produced remissions in some patients with AML, although myelosuppression was again a common adverse effect.[29] The responses in this high-risk population persisted up to 12 months. In addition, patients with non-Hodgkin lymphoma, malignant melanoma, and glioblastoma have been enrolled in clinical trials with other ^213^Bi-labeled compounds, showing its relevant potential for TAT (Table 2).

#### 2.2.3. Astatine-211

^211^At is an α-particle-emitting radionuclide with a physical half-life of 7.2 h and its decay does not result in the production of any relevant daughter isotopes. The first branch decays to ^211^Po (half-life 526 ms), after which it decays through α-particle radiation to stable ^207^Pb. In the second branch, ^211^At α-decays to ^207^Bi, which then results in stable ^207^Pb after emission of X-rays. Theoretically, this offers significant advantages for TAT regarding minimal toxicity and quantitative α-particle emission. However, additional clinical research is needed in order to confirm this as a real advantage. The chemical features of ^211^At are similar to those of iodine, its nearest halogen neighbor, but ^211^At contrarily also tends to behave as a metalloid. Moreover, the exact behavior of ^211^At is far from understood due to the limited knowledge of the chemistry of elemental ^211^At and the lack of any stable equivalent, which excludes the use of conventional analytical techniques for its characterization.[85] Reasonable yields (0.8–2.5 GBq) of ^211^At are obtained via the bombardment of natural bismuth targets with α-particles through the ^209^Bi(α, 2n) ^211^At nuclear reaction in a cyclotron.[86] The 7.2-h half-life of ^211^At is well suited for a multistep synthetic procedure. Consequently, a wide variety of tumor-associated antigens that are aberrantly expressed on the cancer cell surface have been targeted by ^211^At-labeled radiopharmaceuticals.[87,88] To date, ^211^At has been investigated bound to antibodies, thymidine analogs,[89] biotin analogs,[90] colloids,[91] melanin precursors,[92] substrate carriers,[93] and bisphosphonate complexes.[94] Only two clinical studies have been reported so far with ^211^At-labeled molecules.[53,55] The first clinical study for the treatment of recurrent brain tumor provides a proof-of-concept for regional targeted radiotherapy with ^211^At-labeled mAbs.[53] This clinical study demonstrated that the regional administration of ^211^At-ch81C6 was feasible, safe, and resulted in a possible therapeutic benefit for patients with malignant brain tumors. In the second reported clinical study of ^211^At using the MX35 F(ab′)_2_, the compound was delivered successfully through intraperitoneal administration without observed toxicity.[55] These two clinical trials showed no subjective toxicity related to the immunoconjugate and the overall outcomes were highly encouraging. However, there are no clinical data on the toxicity of ^211^At-labeled immunoconjugates after intravenous administration. Further clinical evaluation of ^211^At-labeled compounds in metastatic tumors or residual disease is warranted.

## 3. Vehicles for TAT

The attractive feature of TRNT is its adaptable nature. The radionuclide and the targeting vehicle should in principle be matched to each other in the context of the route of administration, disease stage, target accessibility, and site of action. The selection of both the optimal tumor-associated antigen and the targeting vehicle is a crucial step in the development of a new probe for TRNT. The ideal antigen should be overexpressed on cancer cells, while the expression levels on normal, healthy cells should be extremely low.[95] Examples of biomarkers that are targeted in TAT studies are epidermal growth factor receptor variant III, human epidermal growth factor receptor 2 (HER2), folate receptor alpha, tenascin-C, CD20, CD33, and prostate-specific membrane antigen (Table 2). The vehicle molecules should be optimized to provide a high degree of selectivity and specificity toward the target site or ‘biomarker.’ Below, a section of important vehicles are discussed.

**Figure 1.**
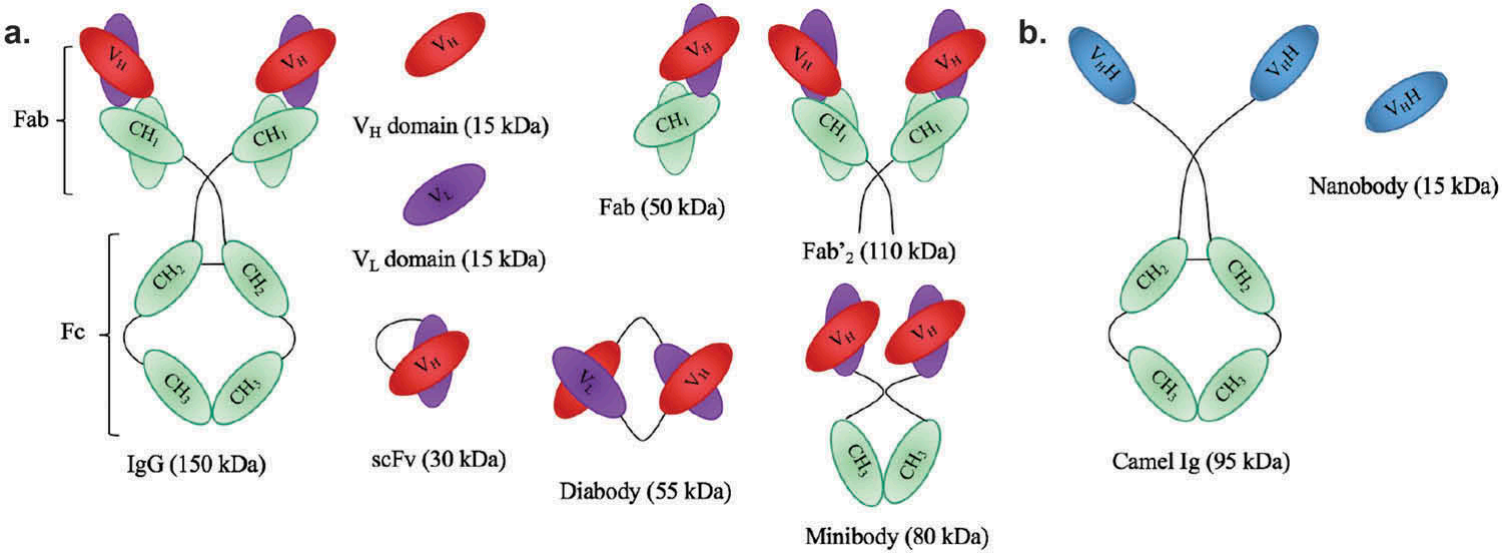
Schematic representation of antibodies and their derived antigen-binding fragments. **a**. Conventional mAb and the derived Fab, scFv, Fv domains V_L_ or V_H_, Fab’_2_, minibody and diabody. **b**. Camelid heavy-chain-only antibody and its V_H_H (also known as nanobody).

mAbs are Y-shaped proteins that contain two identical *fragment antigen-binding* (Fab) fragments and a *fragment crystallizable* (Fc) region (Figure 1). They are produced by plasma cells (mature, activated B cells) and are recruited by the immune system to identify and destroy foreign objects. Moreover, they have the capacity to bind any potential antigen epitope with high affinity, including tumor-associated biomarkers. Today, a variety of preclinical and clinical investigations were conducted using mAbs labeled with α-particle-emitting radionuclides (Table 2). The melanoma trials (Table 2) using ^213^Bi-cDTPA-9.2.27 show that solid tumors can be regressed by TAVAT. Moreover, these clinical results demonstrated that TAVAT for melanoma patients were locally efficacious and nontoxic up to 1.4 mCi. In the ^213^Bi-HuM195 phase I study described above, the authors provided a proof-of-concept for the use of α-particle immunotherapy to treat myeloid leukemia. Although ^213^Bi-HuM195 was well tolerated and 14 (78%) of 18 patients had reductions in the percentage of bone marrow blasts, myelosuppression was seen in all treated patients.[28] Similarly, myelosuppression and liver function abnormalities were observed in a phase I/II trial investigating antileukemic effects of ^213^Bi-HuM195 after partial cytoreductive chemotherapy.[29] These toxicities could be explained by the suboptimal pharmacokinetic properties of mAbs as vehicles for TAT. The high molecular weight of mAbs (150 kDa) and the presence of an Fc-region result in a long serum half-life (several days or weeks) and in interactions with Fc-receptors in myeloid and hepatic sinusoidal cells, resulting in higher bone marrow toxicity and accumulation in the liver. Improvement in antibody engineering has led to the development of antibody fragments that are smaller and devoid of Fc, such as 25-kDa single-chain Fv (scFv), Fab (50 kDa), F(ab′)_2_ (110 kDa), diabodies (55 kDa), and minibodies (80 kDa) without compromising their affinity and specificity (Figure 1).[96] Smaller engineered mAb derivatives are more rapidly delivered to the tumor and mediate more effective tumor penetration. Because of their smaller size and lack of Fc, they are more rapidly cleared from the circulation, which is indirectly proportional to the level of kidney retention. Therefore, their administration results in fast tumor uptake with high tumor-to-background ratios. One study reported the successful conjugation of ^213^Bi to anti-HER2 C6.5 scFv and diabody molecules. However, a lack of tumor-specific therapeutic effect was shown, probably resulting from instability of the scFv and diabody molecules *in vivo*.[46] Here, it was concluded that the physical half-life of 45.6 min of ^213^Bi was too short to allow the systemically administered diabody to specifically localize in an established solid tumor. In a subsequent study, ^211^At was coupled to the stable *N*-succinimidyl-*N*-(4-[^211^At] astatophenethyl) succinamate and subsequently conjugated to the C6.5 diabody (Table 2).[58] Here, the somewhat longer physical half-life of ^211^At matches more closely to the rapid tumor targeting and rather fast systemic clearance of the C6.5 diabody. In the ^211^At-MX35 F(ab′)_2_ phase I trial, therapeutic doses were reached for the treatment of ovarian cancer.[55] However, 50% of the initial activity concentration of this radionuclide remained in the peritoneal fluids 24 h after injection, indicating a higher toxicity risk related to this immunoconjugate.

Besides antibodies and antibody derivatives, ligands (e.g. folate), synthetic protein scaffolds (e.g. affibodies), and substrate analogs (e.g. peptides) can also be used as targeting agents in order to specifically deliver the toxic radionuclide.[97–99] Affibody molecules are small single domain proteins with a molecular weight of 6.5 kDa that are derived from one of the immunoglobulin binding domains of staphylococcal protein A.[100] Previous research demonstrated that affibody molecules can bind to their targets within minutes after administration. The binding kinetics of affibodies are similar to that of nanobodies, but faster than the larger sized mAb and its derived fragments. With regard to TAT, affibody molecules directed against the membrane protein HER2 (Z_HER2:342_ and the bivalent version [Z_HER2:4_]_2_) were radiolabeled with ^211^At using the precursor *N*-succimidyl-*para*(trimethylstannyl)benzoate. Based on preliminary results, the authors concluded that the labeling chemistry needs to be improved before this strategy can be translated to clinical studies.[59]

So far, significant improvements have been made in the development and application of optimized vehicles for TAT. While these preliminary results are promising, there is still considerable room for improvement, mainly in the development of new coupling chemistries and elucidation and optimization of the *in vivo* biodistribution.

## 4. Nanobodies: potential vehicles to specifically deliver toxic α-radiation

**Figure 2.**
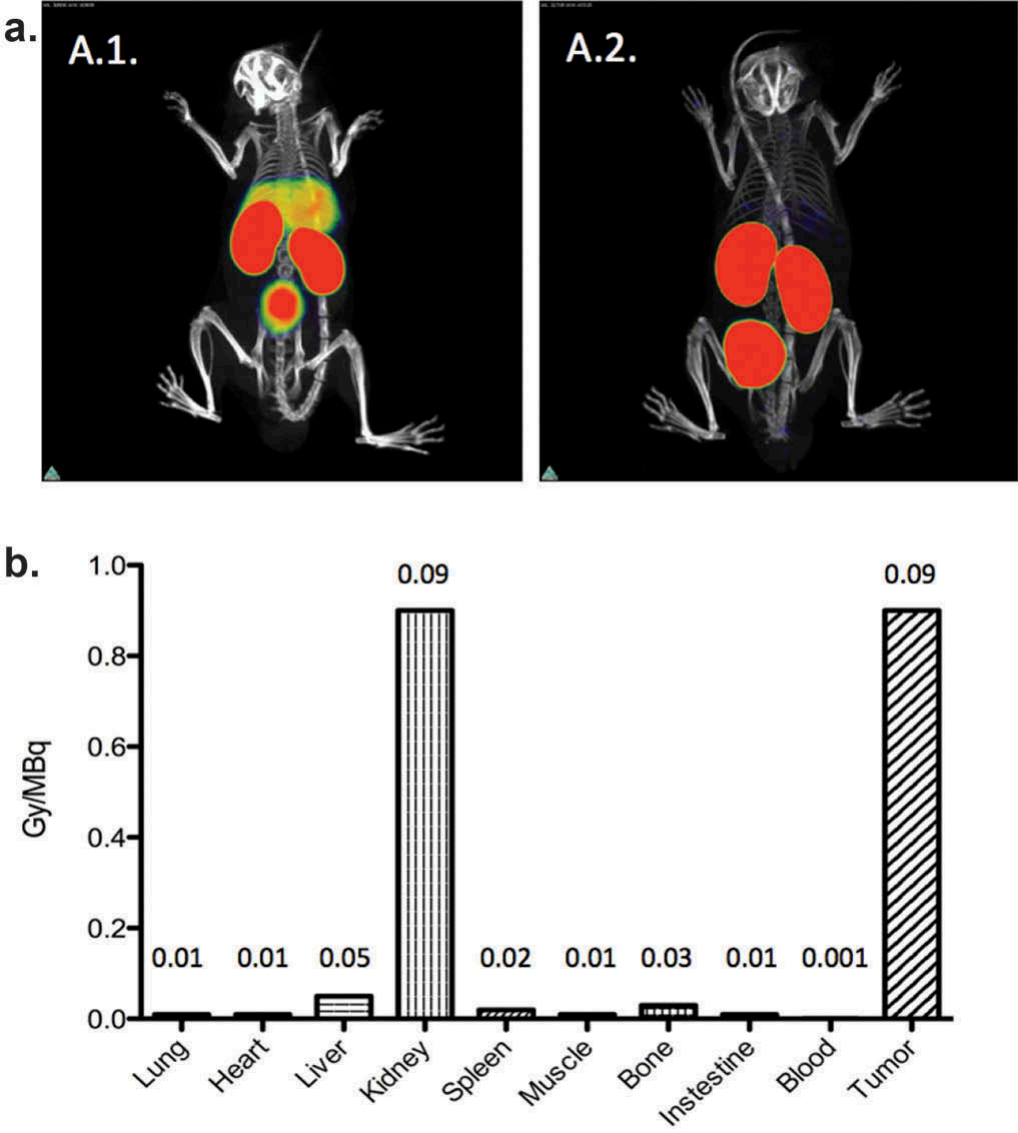
Nanobodies possess numerous advantageous characteristics, including their high antigen specificity (a) and high tumor targeting potential (b). **a**. ^99m^Tc-labeled-nanobody targeting the complement receptor of the Ig superfamily, CRIg, expressed on Kupffer cells in the liver. 3D-rendered SPECT/micro-CT images of naive wild-type (A.1.) and CRIg^−/−^ mice (A.2.) 1 h after intravenous injection of ^99m^Tc-labeled-nanobody. Representative images for 3 mice per group are shown. Figures adapted with permission from.[115] **b**. Dosimetry calculation of untagged ^177^Lu-DTPA-anti-HER2 nanobody coinfused with 150 mg/kg Gelofusin, in HER2^pos^ tumor xenografted mice. Radiolabeling of nanobodies is characterized by significant retention of radioactivity at the kidneys, due to the charged-based aspecific tubular reuptake after glomerular filtration. Figure adapted with permission from.[109].

**Figure 3.**
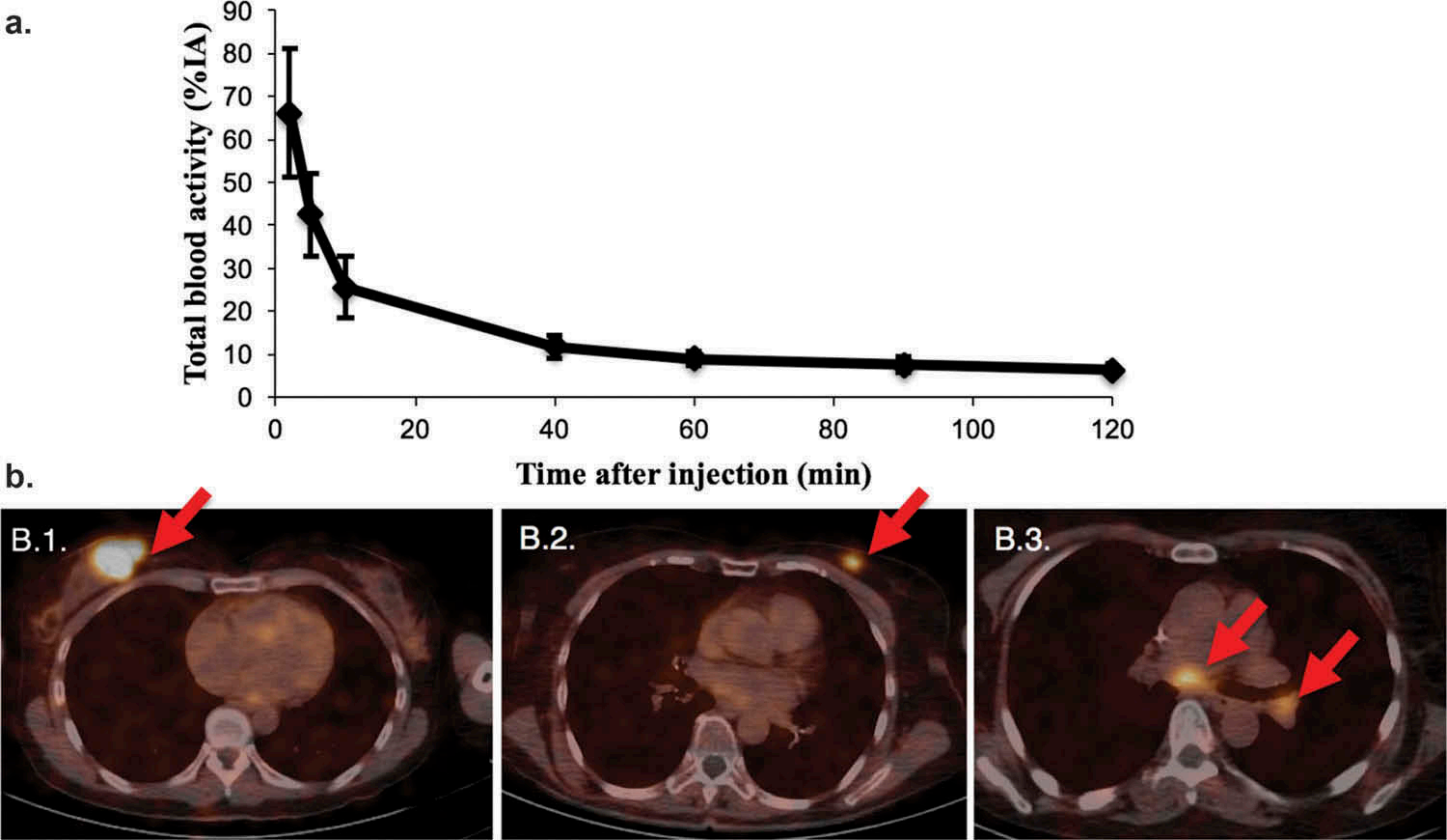
Diagnostic tumor imaging using ^68^Ga-HER2-nanobody in patients with HER2^pos^-breast cancer. **a**. Time-activity curve of total blood activity, expressed in % of injected activity (%IA) (n=20). **b**. Fusion PET/CT images of the uptake of ^68^Ga-HER2-nanobody in breast carcinoma lesions. (B.1.) Patient with the highest tracer uptake (SUVmean 11.8) in a primary breast carcinoma. (B.2.) Patient with moderate tracer uptake in the left breast, which is easily discernable from background (SUVmean 4.9). (B.3.) Patient with invaded lymph nodes in the mediastinum and left hilar region. Lesions are indicated by red arrows. Figures are adapted with permission from.[110].

Recently, there has been a growing interest in the use of nanobodies as vehicles for TRNT. Nanobodies are the smallest, antigen-binding fragments from unique heavy-chain-only antibodies naturally occurring in *Camelidae* (Figure 1).[101] Several applications of nanobodies as *in vivo* diagnostic tracers have been and are currently being developed.[102] Nanobodies have many favorable characteristics as targeted tracers, including high stability in harsh conditions, such as elevated temperatures and extreme pHs offering the potential to use a broader range of radiochemistry methods. Other favorable characteristics include high affinity and specificity for their cognate antigen and facile production (Figure 2(a,b)). As such, nanobodies have been developed as efficient radiotracers directed against a variety of membrane-bound biomarkers [103] in various animal models of cancer,[104–107] inflammation,[108] and cardiovascular diseases [102] using SPECT/PET. Because of their exceptional targeting specificity that is unaffected by labeling with various radionuclides, nanobodies have become valuable vehicles for both nuclear imaging and TRNT.[105–107] Furthermore, nanobodies possess various advantages over mAbs. First, the molecular weight of nanobodies (15 kDa) is one-tenth of that of conventional Abs (150 kDa), making it possible to recognize and bind hidden isotopes. Second, nanobodies have a low immunogenicity because of their rapid blood clearance and high sequence identity to human variable domains of the heavy chain. Furthermore, previous studies by our group demonstrated that nanobodies efficiently penetrate tumor tissues and bind tumor antigens rapidly and specifically *in vivo*. Meanwhile, there is very little nonspecific binding to other tissues, which, along with the rapid blood clearance, results in high tumor-to-background ratios as early as 1 h after injection.[109] Therefore, the nanobody technology could provide an adequate solution to the off-target toxicity problem caused by long blood circulation, as is observed during mAb-based TRNT. A first-in‐human PET study with a GMP-grade HER2-targeting nanobody-based tracer for breast cancer has recently been completed at our university hospital [110] and new clinical trials with nanobodies targeting HER2 and tumor-associated macrophages are planned for 2016. The first clinical study confirmed the fast clearance of nanobodies in patients, with only 10% of the injected activity remaining in the blood at 1 h p.i. (Figure 3(a)). In addition, high tumor-to-background ratios could be observed in 17 out of 19 primary tumors, with mean standard uptake values ranging between 0.7 and 11.8 (Figure 3(b)). Furthermore, the utility of nanobodies as vehicles for TRNT has been investigated in preclinical models using the β^−^-particle-emitting radionuclide ^177^Lu. The most relevant *in vivo* study demonstrated that ^177^Lu-labeled anti-HER2 nanobody efficiently targeted HER2^pos^ s.c. xenografts in a 5-day follow-up study, while radioactivity levels in normal organs were low (Figure 2(b)).[109] Weekly i.v. administrations of ^177^Lu-labeled anti-HER2 nanobody in mice with small HER2^pos^ tumors completely prevented tumor growth, while tumors grew exponentially in untreated mice or in mice receiving a control, nontargeting nanobody. In addition, TRNT using a ^177^Lu-labeled anti-5T2 multiple myeloma nanobody led to an inhibition of disease progression in treated mice compared to control animals.[111] These proof-of-concept TRNT studies show that nanobodies display a more beneficial toxicity profile than mAbs and can deliver a specific lethal radiation dose to a developing tumor. The low molecular weight of nanobodies, below the kidney cut-off for glomerular filtration, and the subsequent charged-based nonspecific tubular reuptake result in significant accumulation and retention of radioactivity in the kidneys. To avoid potential kidney-related toxicities, strategies were tested to reduce renal retention. Both the removal of nonessential positively charged amino acids in the nanobody sequence and co-infusion with positively charged amino acids or the plasma expander Gelofusin were able to lower kidney retention significantly.[107,109] Another approach to reduce the kidney retention is to use optimized radiolabeling procedures. For instance, Zalutsky and colleagues labeled an anti-HER2 nanobody with iodine-131 (^131^I), using the prosthetic group *N*-succinimidyl-4-guanidinomethyl-3-iodobenzoate (SGMIB). SGMIB is a prosthetic group used for antibody and small-protein radioiodination and possesses improved properties as a group that stabilizes ^131^I and maximizes the retention in tumor cells.[112,113] Remarkably, ^131^I-SGMIB-anti-HER2-nanobody was not retained in the kidneys, while tumor targeting was maintained. In addition, Zalutsky and coworkers recently labeled an anti-HER2 nanobody with ^211^At, using this similar residualizing agent, referred to as *N*-succinimidyl-3-[^211^At]astato-4-guanidinomethylbenzoate (SAGMB).[114] Paired-label biodistribution studies directly compared the *in vivo* behavior of ^211^At-SAGMB-nanobody to that of its ^131^I analog SGMIB-nanobody in athymic mice, showing excellent preservation of HER2 binding after ^211^At labeling in combination with high internalization and optimal tumor uptake. Further investigation of this ^211^At-SAGMB-nanobody compound is warranted.

## 5. Conclusion

TAT is an emerging and promising treatment modality that has the ability to specifically kill isolated cancer cells or cell clusters and might only cause little damage to healthy nontarget tumor cells. The combination of preclinical and clinical data affirms the potential of TAT. However, more research is needed to identify the ideal combination of targeting vehicle and α-particle radionuclide, its specific way of linking both, and all this optimized toward specific target expression, disease stage, target accessibility, and site of action.

## 6. Expert opinion: nanobodies coupled to α-particle-emitting radionuclides in cancer therapy

There is an unmet need to treat minimal residual disease and micrometastatic spread of tumor cells, as the current cancer treatment options like chemotherapy, surgery, and external beam radiotherapy are less effective once the tumor has metastasized. Targeted α-particle therapy or TAT allows, due to the high LET of the associated radioactivity, precise delivery of a highly toxic radiation to target cells with reduced harm to normal untargeted cells in the vicinity. This strategy might be ideal for the treatment of small malignant cell populations that are located in the proximity of essential normal tissue structures and could be used in addition to other existing treatment modalities. Increased production and evaluation of α-particle emitters has improved their availability, enhancing the development for new TATs. Currently, TAT has mainly been explored using mAbs. However, the high molecular weight of mAbs and the presence of the Fc-region result in a long serum half-life and interactions with cells containing Fc-receptors. Consequently, the systemic administration of radiolabeled mAbs results in a prolonged presence of radioactivity in blood and highly perfused organs, and unwanted radiation exposure of nontargeted cells. Unsurprisingly, myelotoxicity has been shown to be a limiting factor in several preclinical and clinical studies. Moreover, the dose delivered to carcinomas is often inadequate, owing to the limited penetration of mAb-based vehicles. Hence, we claim that mAbs are not the ideal vehicles to couple with an α-particle emitter. Ab engineering is an interesting approach to overcome some of the limitations of mAbs. Nanobodies in particular have emerged as excellent Ab fragments, as they exhibit high affinity and specificity, fast diffusion and clearance kinetics *in vivo*, high tumor-to-normal-tissue ratios, and a high stability. Moreover, nanobodies have already proven their value in both diagnostic and therapeutic applications. We believe that nanobodies, with their improved properties compared to full-size mAbs and larger Ab-fragments, could be ideal vehicles for TAT.

A key element in the design of radiopharmaceuticals is attuning the properties of the therapeutic radionuclide with those of the tumor-targeting vehicle. The main goal here is to optimize the vehicle in such a way that it fits the characteristics of the α-particle-emitting radionuclide, resulting in optimal tumor targeting and minimal exposure of normal organs. Due to their half-life in the range of minutes to hours, ^213^Bi and ^211^At could be ideal radioactive partners for fast and specific targeting nanobodies. However, both radioisotopes have both their advantages and disadvantages. Currently, the most important limitation of ^211^At is the limited availability of accelerators that are able to generate the 28 MeV α-particle beam required to produce useful levels of ^211^At.[86] Therefore, production and supply of sufficient amounts of ^211^At is still challenging, although over the past few years some progress has been made in the recruitment of new cyclotrons for commercial ^211^At production. Today, about 30 cyclotrons in the world have the beam characteristics (28 MeV) capable for the production of ^211^At. Furthermore, Lindegren and colleagues developed a fully automated procedure that enables automatic, reproducible, rapid, high-yield production of clinically relevant amounts of ^211^At and ^211^At-labeled radiopharmaceuticals.[116] To date, only two clinical trials have been reported using ^211^At-labeled molecules (Table 2). In the first clinical trial, the median survival for patients with glioblastoma multiforme, anaplastic astrocytoma, and oligodendroglioma was 54, 52, and 116 weeks after ^211^At-labeled chimeric anti-tenascin 81C6 therapy.[53] In the second phase I study, ovarian cancer patients were injected with ^211^At-MX35 F(ab′)_2_. Intraperitoneal administration of this immunoconjugate showed that it was possible to achieve therapeutic absorbed doses (15.6 ± 1.0 mGy/[MBq/L]) in the peritoneal peritoneum, where the microscopic tumor clusters are situated, without significant toxicity.[55]

Targeting vehicles can be astatinated via a variety of prosthetic groups.[85] However, many prosthetic groups fail to deliver relevant amounts of astatinated end product, as well as proper *in vivo* stability. In addition, automatable chemistries with high radiochemical yields are yet to be developed. Therefore, many ^211^At-labeled compounds labeled have been abandoned in the past. To this, a more in-depth understanding of the chemistry of ^211^At is required to provide future, useful astatinated radiopharmaceuticals. The production of ^213^Bi is more straightforward, through the actinium-225/bismuth-213 generator system. However, the use of ^213^Bi has been limited by the availability of ^225^Ac. In numerous clinical studies, ^213^Bi (*t*_1/2_ = 46 min) has shown to be effective to treat patients with malignant melanoma, metastatic breast cancer, prostate cancer, pancreatic cancer, and other metastatic diseases. The labeling of targeting vehicles with ^213^Bi is generally performed using straightforward chelating agents such as DTPA and DOTA. In addition, ^213^Bi decays via a branched pathway by α and β emissions to stable ^209^Bi, leading to low toxicity due to the minimal recoil energy the daughter experience upon α-decay. However, the short half-life of ^213^Bi might eventually limit its clinical applicability, as relevant therapeutic doses of ^213^Bi need to be available on a regular basis. Based on these characteristics and on the corresponding features of nanobodies, we claim that nanobodies are ideal for radiolabeling with short-lived radionuclides such as ^211^At and ^213^Bi.

α-Particle recoiling daughter isotopes pose serious problems during TAT as they can do significant harm to healthy tissue when they are not retained at the tumor site. Different approaches to limit the distribution of recoiling daughter isotopes have been found such as encapsulation in a nano-carrier and fast internalization of the α-particle inside the tumor cells. In general, monovalent nanobodies only show limited degree of internalization inside tumor cells after binding. However, it has been shown that internalization can be stimulated by the development of multivalent nanobody constructs, which would augment the amount of α-particles trapped inside the tumor cell.[117]

In order to become valuable, some aspects concerning nanobody-based TAT need to be considered.[1] In general, nanobody targeting is characterized by only moderate absolute uptake in tumor tissue (compared to longer circulating mAbs) and fast blood clearance. To this, it will be important to assess the maximum dose that can be delivered to target tissues. The fast clearance and very specific way of targeting of nanobodies allows repeated injections. In the past, we have shown that therapeutic doses can be reached through fractionated administration using ^177^Lu-labeled nanobodies.[109,111] We therefore believe that therapeutic TAT doses will be achieved by means of repeated administration. The high LET of α-particles will have their beneficial effect on tumor tissues, but can in parallel cause toxicity in tissues with elevated uptake or retention. It is known that nanobodies can interact with the negatively charged lumen of kidney tubuli during filtration from blood. It is therefore of upmost importance to assess the effect of nanobody-TAT at the level of the kidneys. Based on previous published work, there are several countermeasures that can be taken to reduce renal retention of radiolabeled nanobodies. Kidney retention can be reduced significantly by removal of the nanobodies’ amino-acid tag or through co-infusion with the plasma expander Gelofusin and positively charged amino acids.[107,109] In addition, it has been shown that the linker between radioisotope and targeting vehicle can have a dramatic influence on the degree of kidney retention. Recently, anti-HER2 nanobodies were radiolabeled with ^131^I using the prosthetic group SGMIB. It was shown that while the retention in tumor cells was maintained, a complete absence of kidney retention was observed.[112] Interestingly, this exact prosthetic group can be used for astatination of nanobodies. The short path length of α-particles causes a heterogenous distribution in both tumor and tissues, which can lead to very localized toxicity (suborgan or subtissue level). Novel methods that allow micro- and small-scale dosimetry will be essential to realistically estimate dosimetry of TAT-based radiopharmaceuticals. Emerging strategies include the recent development of the α-camera that allows *ex vivo* imaging of α-particle deposits at a cellular level.

In conclusion, the superior characteristics of α-particle emitters ^213^Bi and ^211^At as toxic payload and nanobodies as targeting vehicles offer exciting possibilities in TAT. We therefore expect that the pairing of short-lived α-particle emitters and fast and specific targeting nanobodies will show their potential in the future.

## Article highlights

- Due to the short range in tissue and high linear energy transfer of α-particles, targeted alpha therapy (TAT) is ideal for micrometastatic or residual disease
- Nanobodies are the smallest antibody-derived antigen-binding fragments and have superior characteristics compared to classical mAbs and their derived fragments for *in vivo* cell targeting
- Nanobodies are being evaluated intensively as both diagnostic tracers for nuclear imaging and vehicles for TRNT
- The combination of the short-lived α-particle emitters ^211^At and ^213^Bi and nanobodies offer new possibilities for their application in TAT, which will most likely be demonstrated by ongoing and planned research work.

The box summarizes key points contained in the article.
